# Supplementary material for: Initial In-Hospital Visit-to-Visit Heart Rate Variability Is Associated with Higher Risk of Atrial Fibrillation in Patients with Acute Ischemic Stroke
Source: J Clin Med. 2023 Jan 29;12(3):1050. doi: 10.3390/jcm12031050 (PMC9918220; doi:10.3390/jcm12031050)
Supplement: Supplementary file 1 [file jcm-12-01050-s001.zip › Supplementary Table S1.pdf]

Supplementary Table S1. Demographic and baseline characteristics of the AIS patients stratified by the HR-CV

	HR-CV categories				
	< 0.08	≥ 0.08 and < 0.10	≥ 0.10 and < 0.12	≥ 0.12	
Parameter	N = 2,293	N = 1,649	N = 1,453	N = 2,784	*P* value
Age (years)					< 0.001
Mean (SD)	65.71 (13.26)	67.60 (13.63)	68.42 (13.71)	70.04 (13.38)	
Median (Q1, Q3)	67.00 (57.00, 76.00)	69.00 (59.00, 78.00)	70.00 (60.00, 79.00)	72.00 (61.00, 80.00)	
Male	1399 (61.0)	985 (59.7)	933 (64.2)	1680 (60.3)	0.048
eNIHSS					< 0.001
Mean (SD)	6.25 (4.70)	8.17 (6.71)	8.94 (6.92)	9.84 (7.33)	
Median (Q1, Q3)	4.06 (4.06, 5.66)	4.06 (4.06, 9.43)	4.06 (4.06, 13.07)	5.66 (4.06, 15.48)	
Hypertension	1646 (71.8)	1215 (73.7)	1079 (74.3)	1991 (71.5)	0.148
Diabetes mellitus	937 (40.9)	563 (34.1)	485 (33.4)	852 (30.6)	< 0.001
Dyslipidemia	1117 (48.7)	734 (44.5)	625 (43.0)	1124 (40.4)	< 0.001
Congestive heart failure	131 (5.7)	111 (6.7)	141 (9.7)	304 (10.9)	< 0.001
Coronary artery disease	237 (10.3)	167 (10.1)	159 (10.9)	308 (11.1)	0.719
Current smoker	669 (29.2)	439 (26.6)	393 (27.0)	679 (24.4)	0.002
History of cancer	160 (7.0)	75 (4.5)	81 (5.6)	162 (5.8)	0.014
Body mass index, kg/m^2					0.004
Mean (SD)	24.89 (4.18)	25.01 (4.44)	24.78 (4.10)	24.50 (4.29)	
Median (Q1, Q3)	24.49 (22.06, 27.14)	24.62 (22.11, 27.39)	24.39 (22.01, 27.29)	24.22 (21.60, 26.85)	

Total cholesterol (mmol/L)					< 0.001
Mean (SD)	4.65 (1.10)	4.59 (1.12)	4.52 (1.09)	4.45 (1.07)	
Median (Q1, Q3)	4.58 (3.91, 5.28)	4.48 (3.83, 5.21)	4.43 (3.83, 5.10)	4.35 (3.76, 5.00)	
Triglyceride (mmol/L)					< 0.001
Mean (SD)	1.54 (1.03)	1.46 (1.07)	1.37 (1.01)	1.29 (1.06)	
Median (Q1, Q3)	1.30 (0.92, 1.85)	1.21 (0.87, 1.72)	1.13 (0.79, 1.64)	1.08 (0.79, 1.53)	
Creatinine, μmol/L					< 0.001
Mean (SD)	114.09 (127.41)	114.47 (131.19)	109.44 (101.99)	110.41 (102.06)	
Median (Q1, Q3)	83.10 (67.18, 107.85)	83.98 (68.07, 108.73)	86.63 (70.72, 109.40)	87.52 (71.60, 111.38)	
ALT, U/L					0.123
Mean (SD)	26.42 (21.92)	25.36 (21.72)	25.81 (20.81)	27.37 (58.01)	
Median (Q1, Q3)	21.00 (16.00, 30,00)	20.00 (15.00, 28.00)	21.00 (16.00, 29.00)	21.00 (15.00, 29.00)	
Mean SBP (mmHg)					< 0.001
Mean (SD)	149.58 (19.78)	149.89 (19.48)	148.82 (18.92)	146.20 (18.97)	
Median (Q1, Q3)	147.89 (135.07, 163.08)	149.18 (136.28, 162.73)	148.16 (135.55, 161.72)	144.87 (133.27, 159.00)	
Mean DBP (mmHg)					< 0.001
Mean (SD)	84.41 (10.87)	84.65 (11.32)	84.08 (11.61)	82.80 (11.29)	
Median (Q1, Q3)	83.29 (77.14, 91.00)	84.41 (77.00, 91.71)	83.39 (76.31, 91.57)	82.20 (75.22, 90.17)	
Mean heart rate, bpm					< 0.001
Mean (SD)	74.88 (12.08)	76.12 (12.14)	76.61 (12.13)	76.05 (11.77)	
Median (Q1, Q3)	74.44 (66.93, 80.64)	74.67 (67.92, 82.53)	75.06 (68.32, 84.93)	75.17 (67.45, 83.94)	

Abbreviations: AIS, acute ischemic stroke; HR-CV, coefficient of variation of heart rate; SD, standard deviation; Q, quartile; eNIHSS, estimated National Institute of Health Stroke Scale; ALT, alanine transaminase; SBP, systolic blood pressure; DBP, diastolic blood pressure; bpm, beats per minute..
