# Supplementary figures and images for: Initial In-Hospital Visit-to-Visit Heart Rate Variability Is Associated with Higher Risk of Atrial Fibrillation in Patients with Acute Ischemic Stroke
Source: J Clin Med. 2023 Jan 29;12(3):1050. doi: 10.3390/jcm12031050 (PMC9918220; doi:10.3390/jcm12031050)

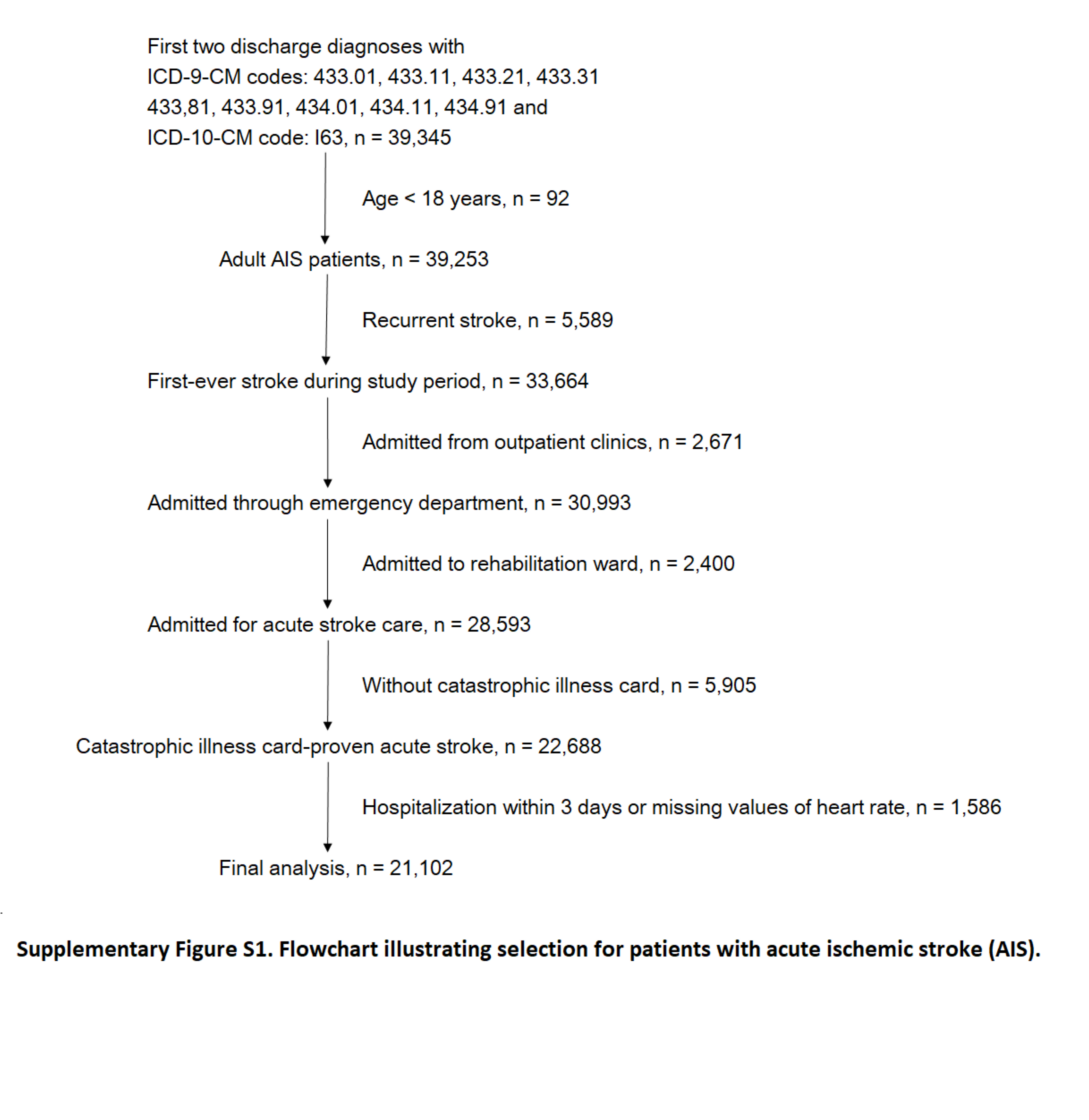

Supplement: Supplementary file 1 [file jcm-12-01050-s001.zip › Supplementary Figure S1.tif]

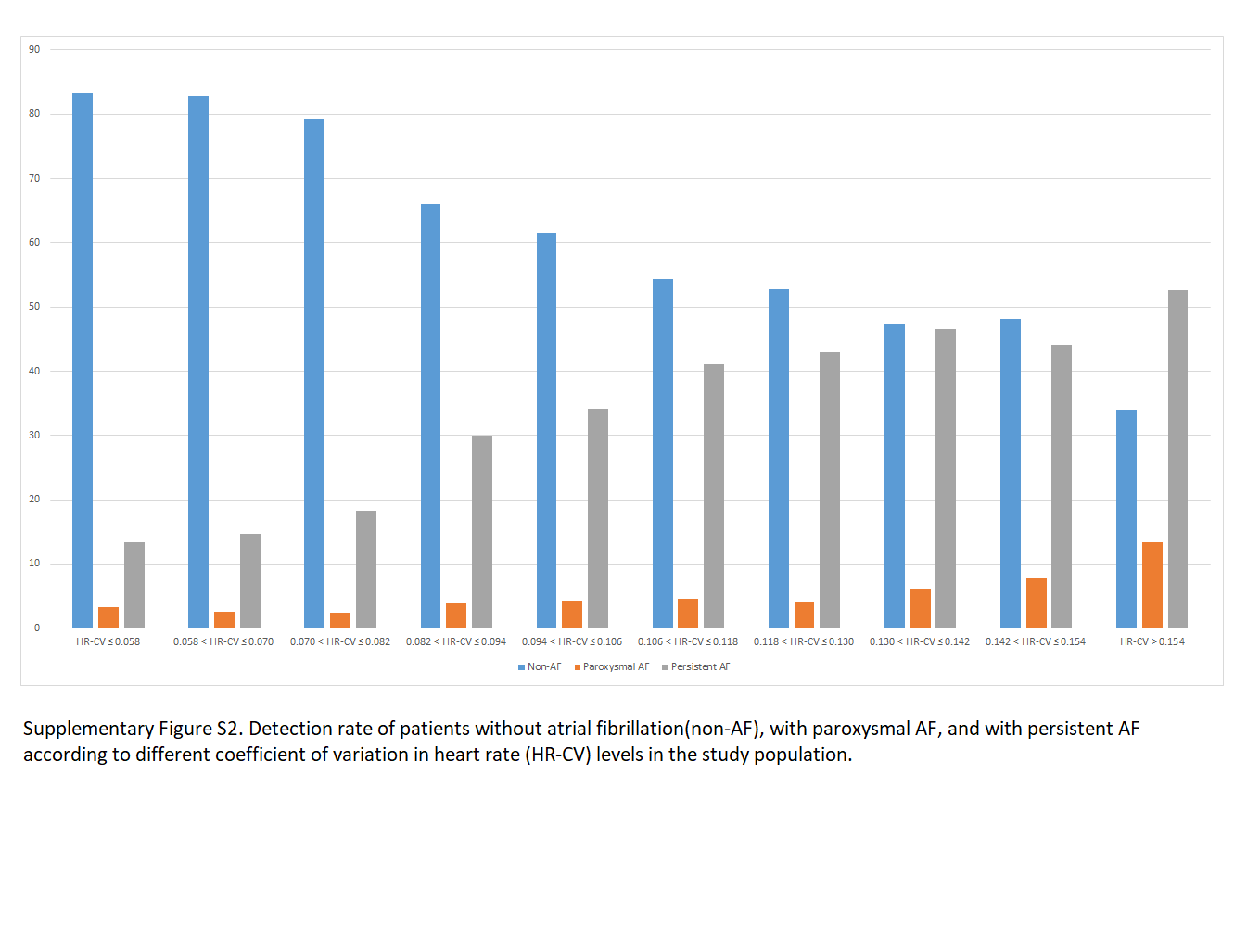

Supplement: Supplementary file 1 [file jcm-12-01050-s001.zip › Supplementary Figure S2_revised.tif]

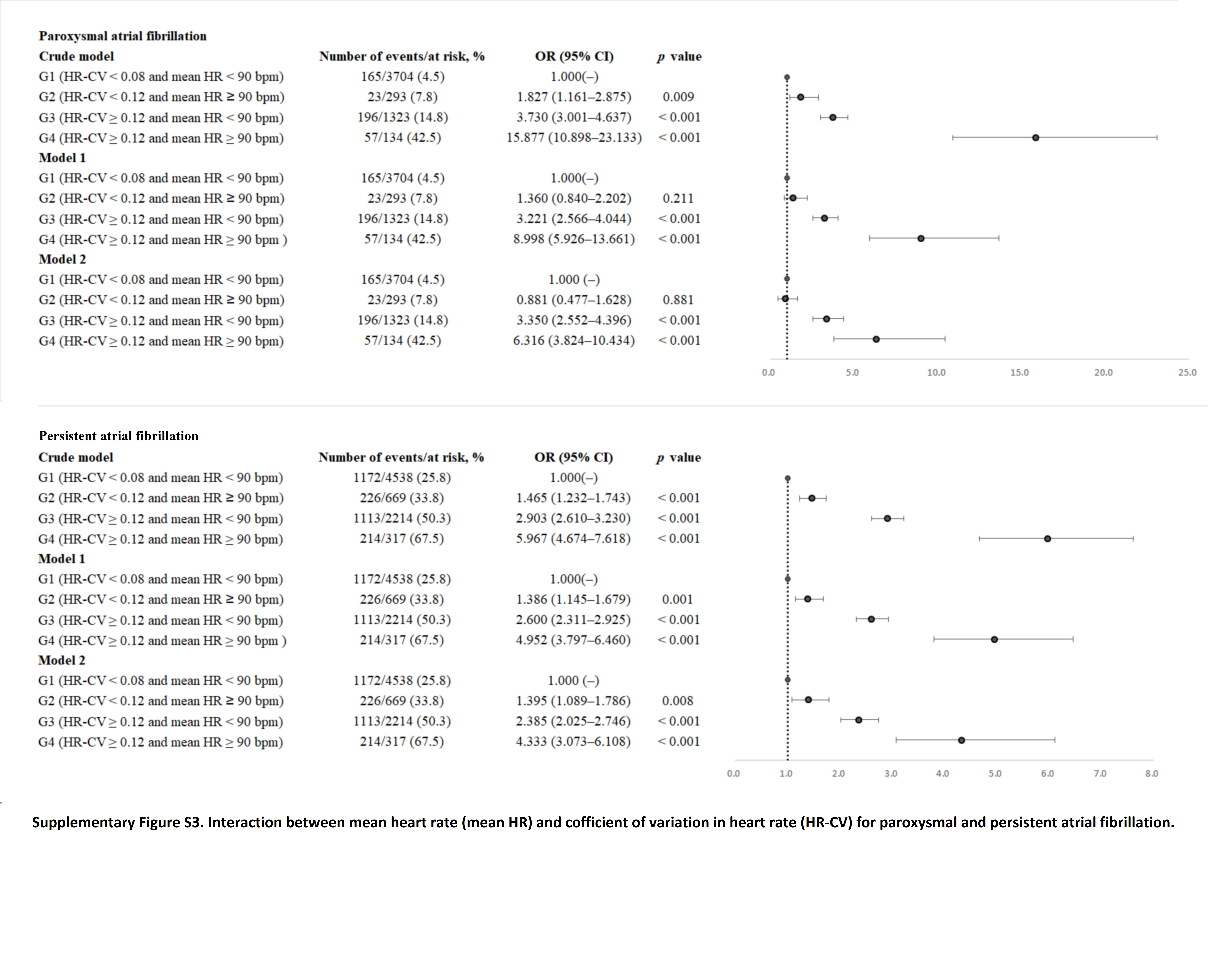

Supplement: Supplementary file 1 [file jcm-12-01050-s001.zip › Supplementary Figure S3_revised.tif]
